# Supplementary figures and images for: Proteomic profiling improves prognostic risk stratification of the Sarculator nomogram in soft tissue sarcomas of the extremities and trunk wall
Source: Cancer Med. 2024 Jul 23;13(14):e70026. doi: 10.1002/cam4.70026 (PMC11263812; doi:10.1002/cam4.70026)

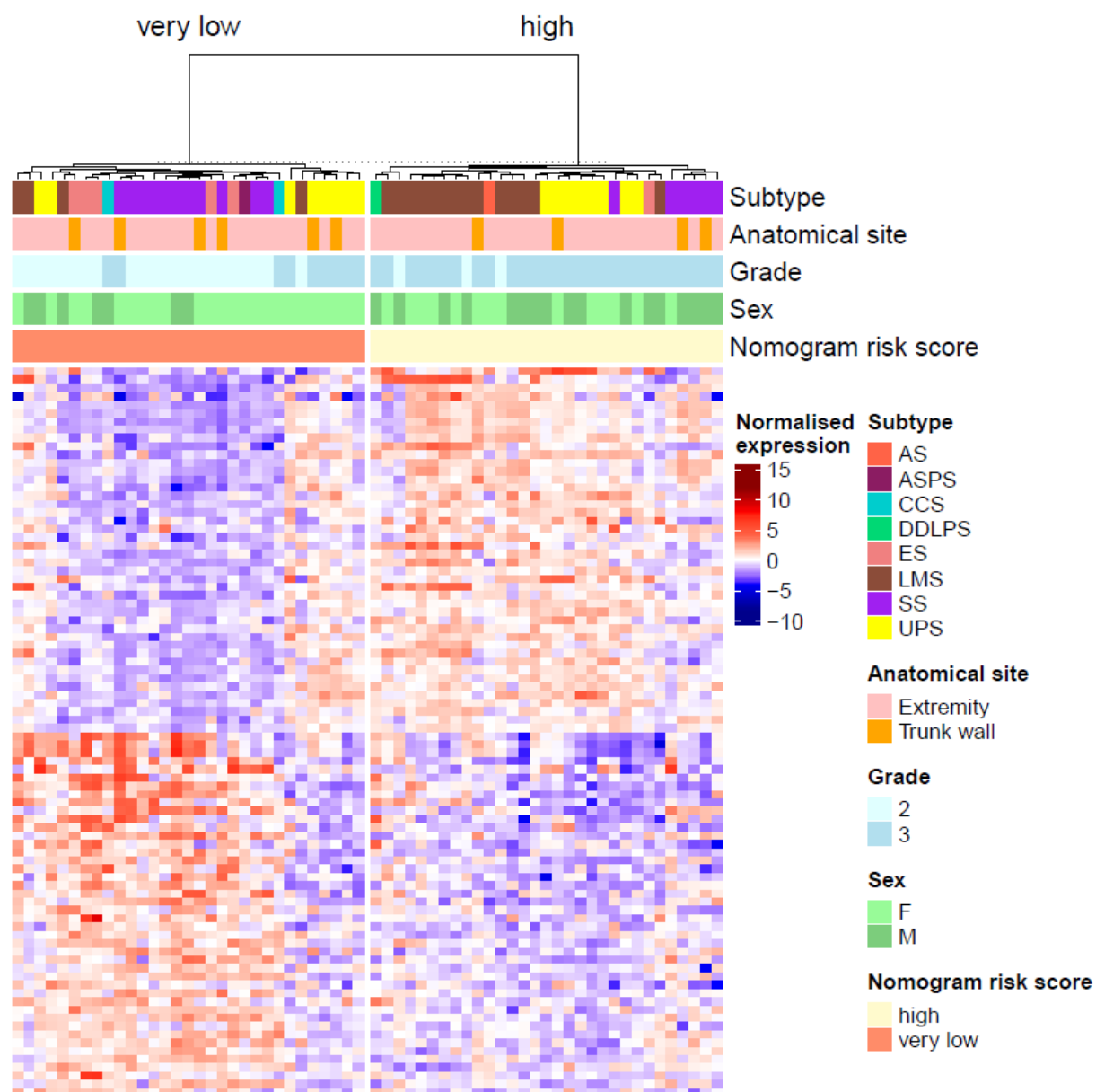

Supplement: Supplementary file 1 — Figure S1. [file CAM4-13-e70026-s005.pdf]

A

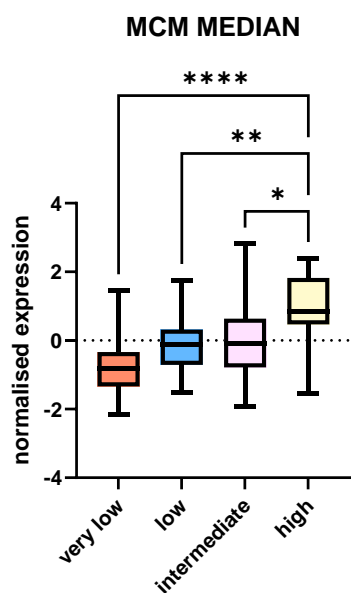

B

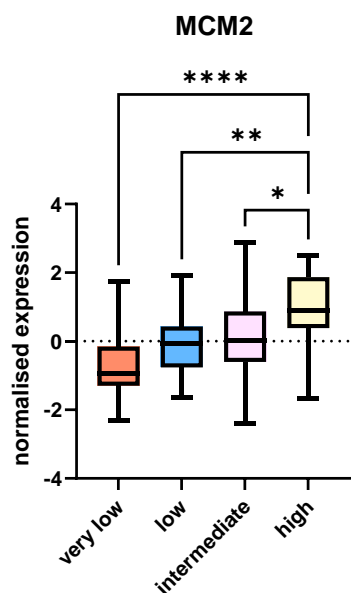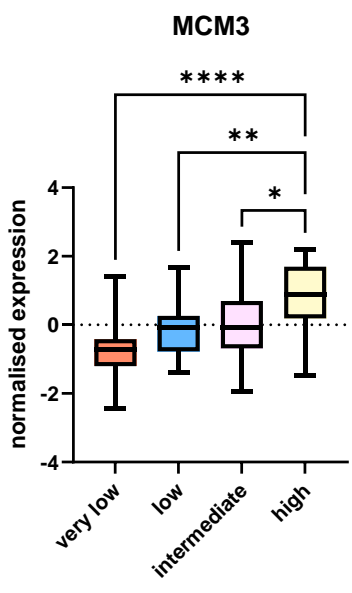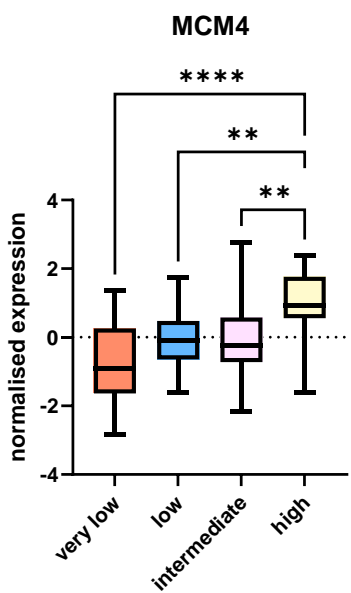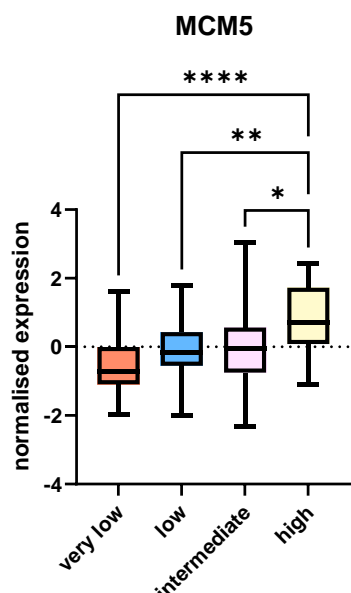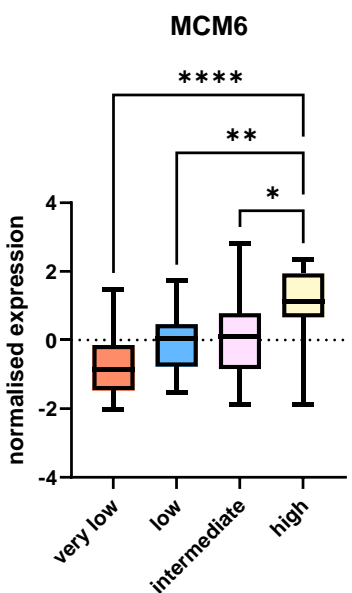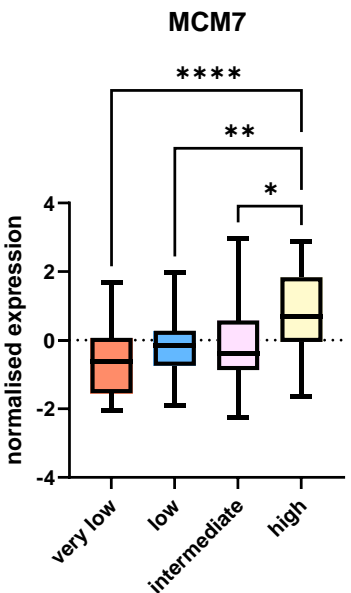

Supplement: Supplementary file 2 — Figure S2. [file CAM4-13-e70026-s004.pdf]

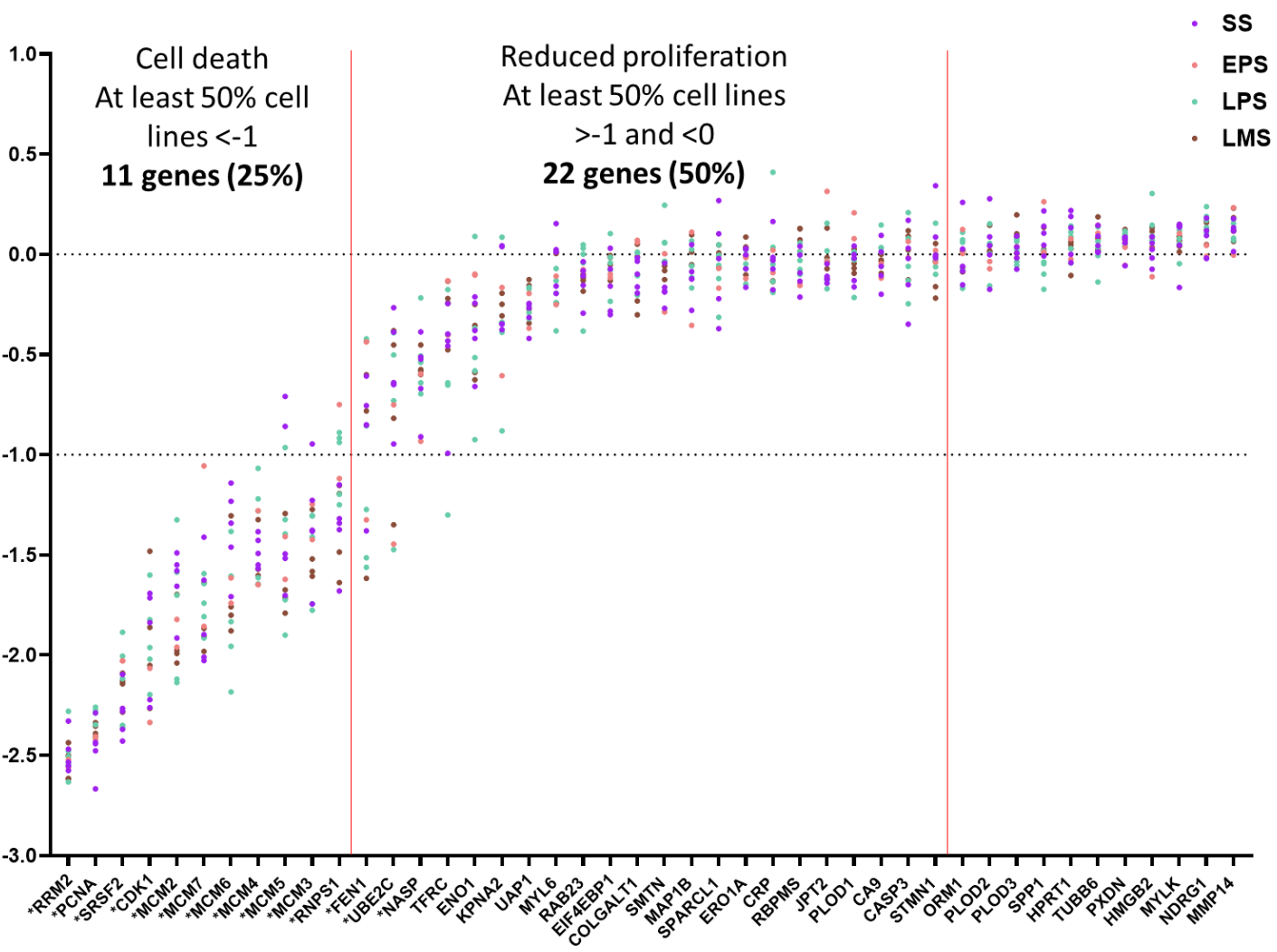

Supplement: Supplementary file 3 — Figure S3. [file CAM4-13-e70026-s003.pdf]

A

Proteomics cohort

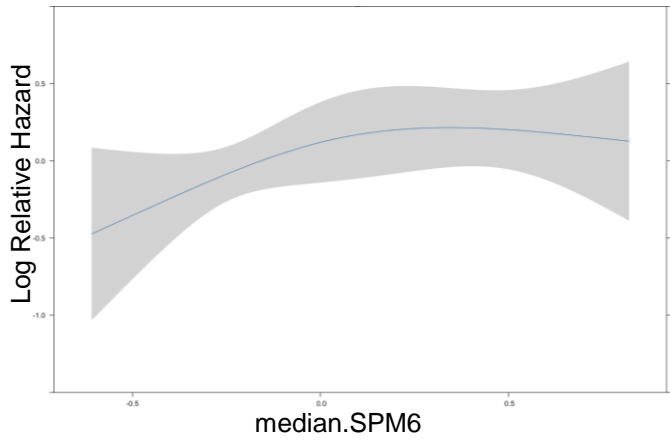

B

TCGA cohort

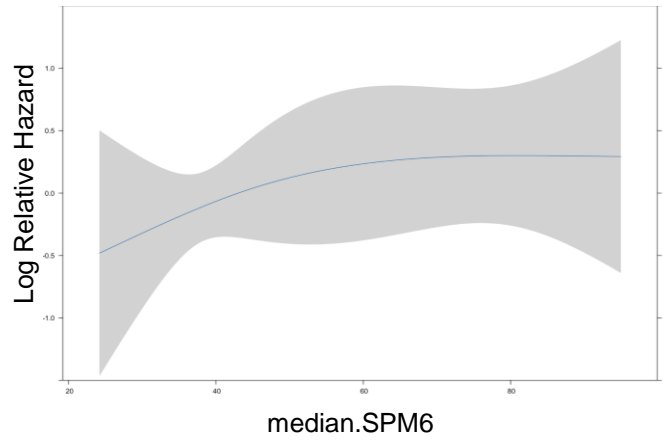

C

Proteomics cohort

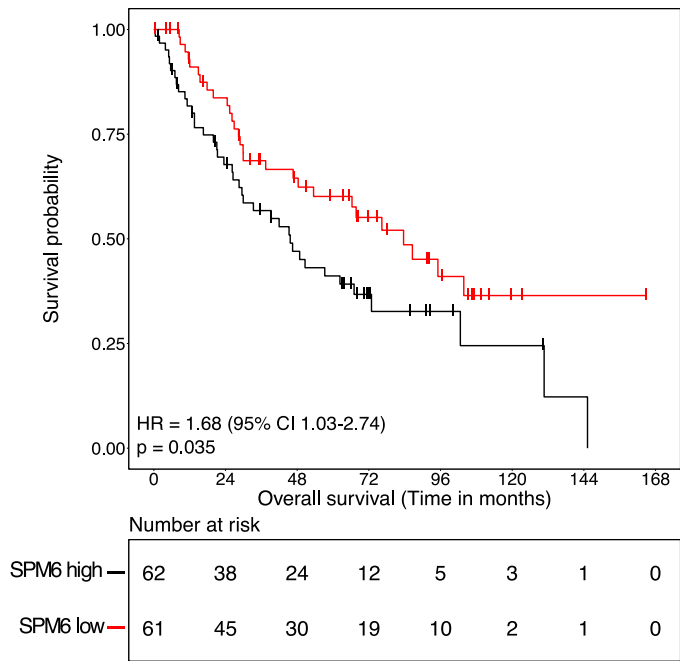

D

TCGA cohort

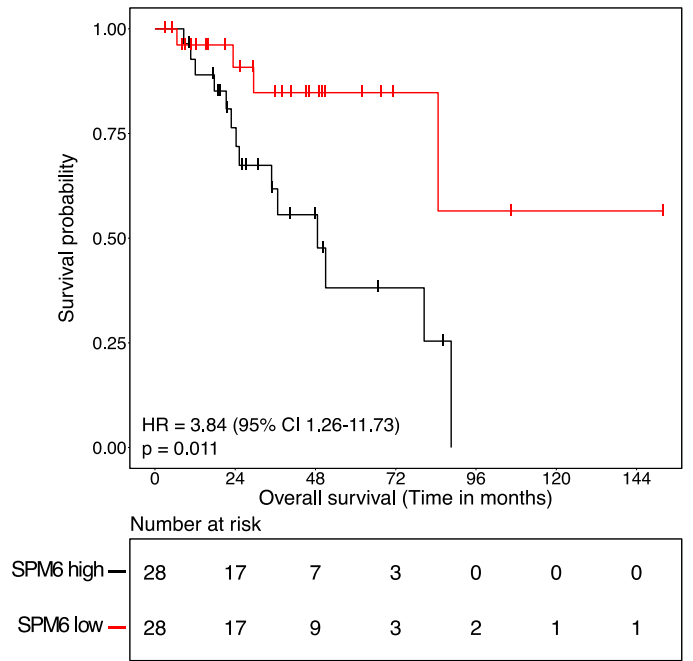

Supplement: Supplementary file 4 — Figure S4. [file CAM4-13-e70026-s008.pdf]
