# Supplementary material for: Proteomic profiling improves prognostic risk stratification of the Sarculator nomogram in soft tissue sarcomas of the extremities and trunk wall
Source: Cancer Med. 2024 Jul 23;13(14):e70026. doi: 10.1002/cam4.70026 (PMC11263812; doi:10.1002/cam4.70026)
Supplement: Supplementary file 9 — Table S5. [file CAM4-13-e70026-s011.docx]

**Table S5.** Univariable Cox regression analysis with Wald test assessing overall survival (OS) for patients in our proteomics cohort stratified based on the Sarculator nomogram risk groups and median expression of Sarcoma Proteomic Module 6 (SPM6). HR=hazard ratio; CI= Confidence interval.

|  | **HR 95% CI p value** |
| --- | --- |
| **Sarculator nomogram**  3^rd^ vs 1^st^ quartile | 2.50 1.77-3.52 <0.0001 |
| **SPM6**  3^rd^ vs 1^st^ quartile | 1.43 0.94-2.19 0.2422 |
