# Supplementary material for: Proteomic profiling improves prognostic risk stratification of the Sarculator nomogram in soft tissue sarcomas of the extremities and trunk wall
Source: Cancer Med. 2024 Jul 23;13(14):e70026. doi: 10.1002/cam4.70026 (PMC11263812; doi:10.1002/cam4.70026)
Supplement: Supplementary file 10 — Table S6. [file CAM4-13-e70026-s001.docx]

**Table S6.** Univariable Cox regression analysis with Wald test assessing overall survival (OS) for STS patients from The Cancer Genome Atlas Consortium (TCGA) stratified based on the Sarculator nomogram risk groups and median expression of Sarcoma Proteomic Module 6 (SPM6). HR=hazard ratio; CI= Confidence interval.

|  | **HR 95% CI p value** |
| --- | --- |
| **Sarculator nomogram**  3^rd^ vs 1^st^ quartile | 2.43             1.19-4.94                0.0144 |
| **SPM6**  3^rd^ vs 1^st^ quartile | 1.59             0.66-3.86                0.5543 |

* Missing tumor size in 2 patients: imputed with median value
