# Supplementary material for: Proteomic profiling improves prognostic risk stratification of the Sarculator nomogram in soft tissue sarcomas of the extremities and trunk wall
Source: Cancer Med. 2024 Jul 23;13(14):e70026. doi: 10.1002/cam4.70026 (PMC11263812; doi:10.1002/cam4.70026)
Supplement: Supplementary file 11 — Table S7. [file CAM4-13-e70026-s002.docx]

**Table S7.** Multivariable Cox regression analysis with two-sided Wald test assessing interaction between Sarcoma Proteomic Module 6 (SPM6) signature and tumour size. HR=hazard ratio; CI= Confidence interval.

|  | **HR 95% CI** |
| --- | --- |
| **Tumour size 1^st^ quartile (5.35 cm)**  **SPM6** | 1.504 0.77-3.09 |
| **Tumour size 2^nd^ quartile (8 cm)**  **SPM6** | 0.92 0.55-1.54 |
| **Tumour size 3^rd^ quartile (11 cm)**  **SPM6** | 0.87 0.48-1.58 |

Wald test p-value (p): interaction tumour size-SPM6 p=0.038; SPM6: linear term p=0.034; non-linear term p=0.008; tumour size: linear term p<0.0001; non-linear term p=0.07.
